# β-Adrenergic Receptor-PI3K Signaling Crosstalk in Mouse Heart: Elucidation of Immediate Downstream Signaling Cascades

**DOI:** 10.1371/journal.pone.0026581

**Published:** 2011-10-19

**Authors:** Weizhi Zhang, Naohiro Yano, Minzi Deng, Quanfu Mao, Sunil K. Shaw, Yi-Tang Tseng

**Affiliations:** 1 Department of Cardiothoracic Surgery, The Second Xiangya Hospital, Central South University, Changsha, Hunan, China; 2 Department of Pediatrics, Women and Infants Hospital, The Warren Alpert Medical School of Brown University, Providence, Rhode Island, United States of America; University of Illinois at Chicago, United States of America

## Abstract

Sustained β-adrenergic receptors (βAR) activation leads to cardiac hypertrophy and prevents left ventricular (LV) atrophy during LV unloading. The immediate signaling pathways downstream from βAR stimulation, however, have not been well investigated. The current study was to examine the early cardiac signaling mechanism(s) following βAR stimulation. In adult C57BL/6 mice, acute βAR stimulation induced significant increases in PI3K activity and activation of Akt and ERK1/2 in the heart, but not in lungs or livers. In contrast, the same treatment did not elicit these changes in β_1_/β_2_AR double knockout mice. We further showed the specificity of β_2_AR in this crosstalk as treatment with formoterol, a β_2_AR-selective agonist, but not dobutamine, a predominantly β_1_AR agonist, activated cardiac Akt and ERK1/2. Acute βAR stimulation also significantly increased the phosphorylation of mTOR (the mammalian target of rapamycin), P70S6K, ribosomal protein S6, GSK-3α/β (glycogen synthase kinase-3α/β), and FOXO1/3a (the forkhead box family of transcription factors 1 and 3a). Moreover, acute βAR stimulation time-dependently decreased the mRNA levels of the muscle-specific E3 ligases atrogin-1 and muscle ring finger protein-1 (MuRF1) in mouse heart. Our results indicate that acute βAR stimulation *in vivo* affects multiple cardiac signaling cascades, including the PI3K signaling pathway, ERK1/2, atrogin-1 and MuRF1. These data 1) provide convincing evidence for the crosstalk between βAR and PI3K signaling pathways; 2) confirm the β_2_AR specificity in this crosstalk *in vivo*; and 3) identify novel signaling factors involved in cardiac hypertrophy and LV unloading. Understanding of the intricate interplay between β_2_AR activation and these signaling cascades should provide critical clues to the pathogenesis of cardiac hypertrophy and enable identification of targets for early clinical interaction of cardiac lesions.

## Introduction

β-adrenergic receptors (βAR) are members of the G protein-coupled receptor family [1]. Cardiomyocytes express all three βAR subtypes- β_1_AR, β_2_AR, and, in some species, β_3_AR [2], [3], [4]. Different subtypes of βAR have the capacity to activate multiple intracellular signaling pathways through different G proteins and second messengers, thereby mediating diverse heart functions under various conditions [5], [6], [7]. There is accumulating evidence that sustained βAR activation leads to cardiac hypertrophy, which represents an independent risk factor for cardiovascular morbidity and mortality [8]. Thus, βAR blockers have become an established treatment of chronic heart failure. On the other hand, several lines of evidence suggest that selective βAR stimulation may be beneficial in the setting of heart failure [9], [10]. More recently, clinical and experimental studies have shown that βAR activation may prevent left ventricular (LV) atrophy during LV unloading [11], [12]. Hence, understanding the signaling mechanisms under which βAR exert diverse roles in different cardiac conditions is likely to provide a better therapeutic approach towards treatment of heart failure. Our knowledge about the mechanisms by which βAR activation promotes myocardial growth was mostly acquired from *in vitro* systems or based on studies using animal models of chronic systemic βAR agonist administration [8]. The immediate signaling pathway(s) following acute βAR stimulation have not been well investigated.

PI3K signaling contributes to many important cellular processes, including regulation of cell cycle, growth, survival and migration [13]. Several genetic models with alterations of PI3K and other signaling molecules, including phosphatase and tensin homologue deleted from chromosome 10 (PTEN) and Akt, have demonstrated altered cardiac phenotypes [14], [15], [16], [17]. Moreover, we have previously demonstrated that the activity of the PI3K signaling pathway during cardiac development is highly regulated with the highest levels found during the fetal-neonatal transition period and the lowest levels in the adult [18].

βAR stimulation has been shown to affect insulin signaling via PI3K/Akt in cardiomyocytes [19]. In cultured cardiomyocytes, βAR stimulation increases PI3K activity [20], [21]. Both the β_1_AR and β_2_AR have been reported to transactivate PI3K *in vitro* [22], [23]. Moreover, βAR stimulation-induced increases in heart weight, contractile abnormalities, and myocardial fibrosis, and cardiac “fetal” genes were markedly attenuated in PI3Kγ-knockout mice [24]. Our laboratory has shown that βAR-induced transactivation of PI3K signaling pathway plays important roles in the regulation of cell proliferation and cellular protection against apoptosis in neonatal cardiomyocytes [25], [26]. Recently, we further provided *in vitro* evidence for such βAR-PI3K crosstalk and identified some critical signaling molecules involved [7]. The crosstalk between βAR and PI3K signaling pathway *in vivo*, however, has not been well studied, especially in a cardiac context. Moreover, the early signaling events following βAR stimulation preceding the onset of cardiac hypertrophy has not been demonstrated. A more detailed picture of these early signaling events would enable identification of targets for early clinical interaction for the βAR-related cardiac lesions.

To elucidate the cardiac signaling mechanisms responsible for the crosstalk between βAR and PI3K signaling pathway after acute βAR agonist administration, wild type adult male C57BL/6 and β_1_/β_2_AR double knockout (β_1_/β_2_ DKO) mice were employed. We demonstrated that multiple signaling molecules downstream of PI3K are affected following acute βAR stimulation, including Akt, P70S6/mTOR/S6 axis, GSK3α/β and FOXOs. More important, two novel players, atrigin-1 and MuRF1, were shown to be part of the early signaling cascade.

## Materials and Methods

### Ethics Statement

This study was carried out in strict accordance with the recommendations in the Guide for the Care and Use of Laboratory Animals of the National Institutes of Health. The protocol was approved by the Institutional Animal Care and Use Committee of the Lifespan (Assurance Number: A3922-01; Protocol Number: 002809). All efforts were made to minimize suffering.

### Materials

Isoproterenol (ISO), a nonspecific βAR agonist, and dobutamine, a predominantly β_1_AR agonist [27], were obtained from Hospira Inc., Lake Forest, IL. Formoterol, a β_2_AR-selective agonist (displays a 330-fold selectivity for β_2_AR over β_1_AR, pK_d_ values are 8.12 and 5.58 respectively), was obtained from Tocris Bioscience, Ellisville, MO. LY294002, a potent inhibitor of PI3K was purchased from Santa Cruz Biotechnology, inc. (sc-201426). All other chemicals and reagents were obtained from Sigma unless stated otherwise.

### Experimental animals

Three months old male C57BL/6 wild-type mice (The Jackson Laboratory, Bar Harbor, ME) and β_1_/β_2_ DKO mice (Strain Name: STOCK Adrb1^tm1Bkk^ Adrb2^tm1Bkk^/J, Stock number: 003810, The Jackson Laboratory) were used. Animals were fed standard laboratory chow ad libitum. Mice were injected intraperitoneally (i.p.) with ISO (1.25 mg/kg), dobutamine (1.7 mg/kg, the same molar equivalent with ISO), formoterol (2.1 mg/kg, the same molar equivalent with ISO) or saline for 30 min (unless otherwise specified) before euthanized as previously described [7]. In some experiments, mice were pretreated with DMSO (5%), LY294002 (1.4 mg/kg, i.p.) or H-89 (20 mg/kg, i.p.), a PKA inhibitor, for 30 min before the ISO treatment described above. Whole hearts were harvested and LV was identified, dissected, flash frozen in liquid nitrogen, and stored at −80°C. Left ventricular tissue lysates were prepared as described [7].

### PI3K Assays

Left ventricular lysates were prepared as described, and protein concentrations were determined with the bicinchoninic acid assay [7], [18]. PI3K activity was determined with *in vitro* immunoprecipitation lipid kinase assay as described previously [7], [18]. Briefly, left ventricular lysates (0.5 mg unless noted otherwise) were immunoprecipitated (IP) with anti-phosphotyrosine (pY) antibody (Upstate, Charlottesville, VA), and L-α-phosphoinositide (Avanti Polar Lipids, Alabaster, AL) was used as the lipid substrate (2 µg/reaction), and converted to phosphoinositide 3-phosphate (PIP). After incubation with γ-^32^P ATP (PerkinElmer, Inc., Boston, MA), the final extracted reaction mixtures were spotted onto silica gel-coated TLC plates (Whatman, Florham Park, NJ) and run in TLC buffer (65% *n*-propanol, 0.54M acetic acid). The results were analyzed by phosphorimaging (Bio-Rad Laboratories).

### Western Blotting

Protein expression was evaluated by Western blotting as previously described [26]. Protein samples were loaded and run in either 4–12% Bis-Tris precast gels or 3–8% Tris-Acetate precast gels (Invitrogen, Carlsbad, CA), blotted onto a PVDF membrane (Bio-Rad, Hercules, CA), and detected by the following antibodies: phospho-Akt (Ser473) antibody (R&D systems, Minneapolis, MN), phospho-Akt (Thr308), total Akt, phospho-ERK1/2 (Thr202/Tyr204), total ERK1/2, phospho-mTOR (Ser2448), total mTOR, phospho-P70S6K (Thr389, Thr421/Ser424), total P70S6K, phospho-S6 (Ser235/236, Ser240/244), total S6, phospho-GSK-3α (Ser21), total GSK-3α, phospho-GSK-3β (Ser9), total GSK-3β, phospho-FOXO1(Thr24)/FOXO3a(Thr32), phospho-FOXO3a (Ser318/321, Ser253), total FOXO1 antibodies (Cell Signaling, Danvers, MA), total FOXO3a and Actin antibodies (Millipore, Billerica, MA).

### Real Time Quantitative-PCR

Total RNA was extracted from ventricular tissue using TRIzol reagent (Invitrogen, Carlsbad, CA), and further purified using RNeasy MinElute Cleanup Kit (Qiagen, Hilden, Germany). RNA concentration and purity were determined by spectrophotometry (Thermo Scientific, Wilmington, DE). The reverse transcription was performed using the Superscript III (Invitrogen) and Oligo (dT) primers from total RNA (1 µg). Quantitative determination of muscle-specific E3 ligases atrogin-1 (atrogin-1) and muscle ring finger protein-1 (MuRF1) mRNA levels was performed by real-time quantitative-PCR using specific TaqMan probes and GAPDH as the endogenous control. The following primer/probe pairs were used: atrogin-1, forward, 5′-ACC AAA ACT CAG TAC TTC CAT CAA GA-3′, reverse, 5′-TGT TGA AAG CTT CCC CCA AA-3′, probe,5′-FAM-CAA AGG AAG TAC GAA GGA GCG CCA TG-TAMRA-3′; MuRF1, forward, 5′-ACA CAA CCT CTG CCG GAA GT-3′, reverse, 5′-ACG GAA ACG ACC TCC AGA CA-3′, probe, 5′-FAM-AGG CTG CGA ATC CCT ACT GGA CCA A-TAMRA-3′; GAPDH, forward, 5′-ATG TTC CAG TAT GAC TCC ACT CAC G-3′, reverse, 5′-GAA GAC ACC AGT AGA CTC CAC GAC A-3′, probe, 5′-FAM-AAG CCC ATC ACC ATC TTC CAG GAG CGA GA-TAMRA-3′ [28]. Real Time Quantitative PCR was performed on a 7500 Fast RT-PCR System (Applied Biosystems) using the TaqMan Gene Expression Master Mix (Applied Biosystems). The mRNA level was determined with the comparative Ct method (2^-ΔΔCt^). Each sample was analyzed in triplicate.

### Statistical Analysis

All data were expressed as mean ± S.E. based on data derived from multiple independent experiments. The intensity of bands from Western blots was scanned with densitometry and digitally analyzed using the Image J software (NIH). The statistical significance was tested by Student's *t*-test. A probability of p<0.05 was considered statistically significant.

## Results

### Acute βAR stimulation induces increases in PI3K activity and phosphorylation of Akt and ERK1/2 in adult mouse heart

**Figure 1:**
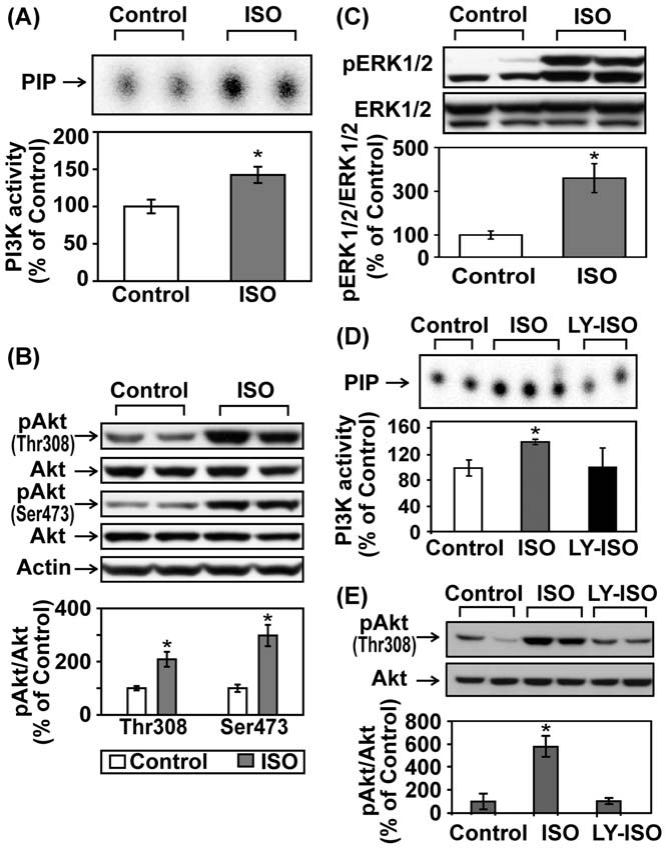
Acute βAR stimulation in vivo increases cardiac PI3K activity and phophorylation of Akt and ERK1/2. Adult male C57BL/6 mice were treated with vehicle control or isoproterenol (ISO, 1.25 mg/kg, i.p.) for 30 min. (A) Left ventricular tissue lysates were IP with an anti-pY antibody and subjected to *in vitro* lipid kinase assay. PIP, the phosphorylated end-product. The bar graph shows the densitometric scanning results of the measurement of PI3K activities in the control (n = 6) and ISO-treated (n = 6) mice. (B, C) Representative Western blot analyses were performed on LV tissue lysates with antibodies against phospho-Akt (Thr308), phospho-Akt (Ser473), total Akt, phospho-ERK1/2 (Thr202/Tyr204) and total ERK1/2. The bar graphs show the densitometric scanning results from two individual experiments (n = 6). In another series of experiments, mice were pretreated with vehicle (5% DMSO) or LY294002 (LY, 1.4 mg/kg, i.p.) for 30 min before the ISO treatment. In vitro lipid kinase assay (D) and Western blot analyses (E) were performed as described above. The bar graph shows the densitometric scanning results in the control (n = 6), ISO (n = 6), and LY/ISO (n = 6) groups. In all Western blotting experiments, data were normalized with individual total protein levels. *, p<0.05 versus vehicle control.

**Figure 2:**
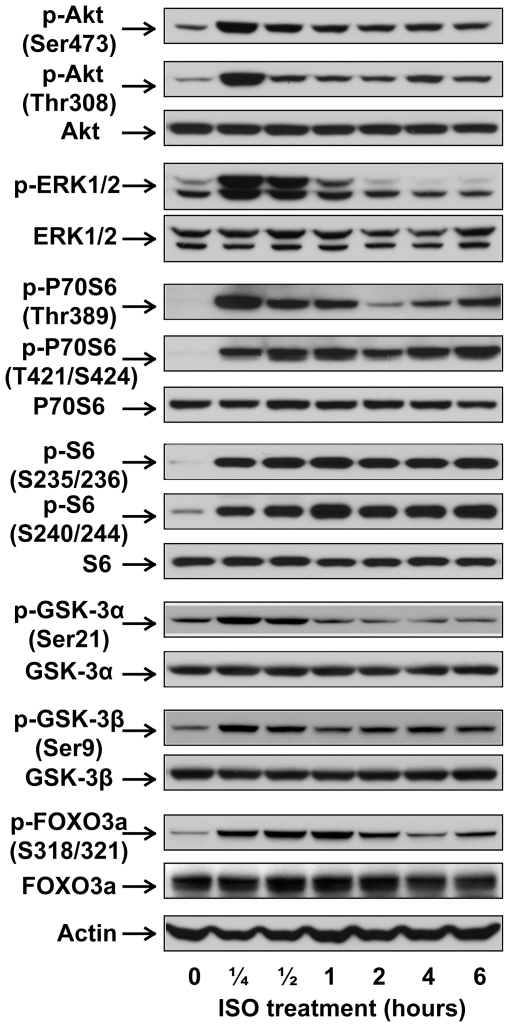
The time course of the effect of βAR stimulation in vivo on phophorylation of Akt, ERK1/2, P70S6K, S6, GSK-3α/β and FOXO3a. Adult male C57BL/6 mice were treated with vehicle control or isoproterenol (ISO, 1.25 mg/kg, i.p.) for the indicated time. Shown are representative Western blots performed on LV tissue lysates with antibodies against phospho-Akt (Thr308), phospho-Akt (Ser473), phospho-ERK1/2 (Thr202/Tyr204), phospho-P70S6K (Thr389), phospho-P70S6K (Thr421/Ser424), phospho-S6 (Ser235/236), phospho-S6 (Ser240/244), phospho-GSK-3α (Ser21), phospho-GSK-3β (Ser9), and phospho-FOXO3a (Ser318/321). Blots of individual total protein and actin were also included.

**Figure 3:**
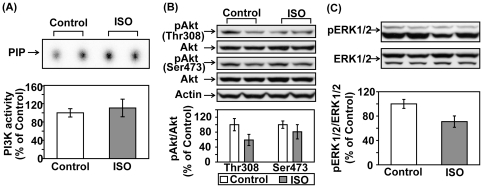
Acute βAR stimulation doesn't affect the activity of PI3K/Akt or ERK1/2 in β1/β2DKO mouse heart. Adult male β_1_/β_2_ double knockout mice were treated with vehicle control or isoproterenol (ISO, 1.25 mg/kg, i.p.) for 30 min. (A) Left ventricular lysates were subjected to *in vitro* lipid kinase assay as described above. The bar graph shows the densitometric scanning results of the measurement of cardiac PI3K activities in the control (n = 3) and ISO-treated (n = 3) mice. (B, C) Representative Western blot analyses were performed on LV tissue lysates as described above. The bar graphs show the densitometric scanning results from three seperate experiments. Data are normalized with individual total protein levels.

**Figure 4:**
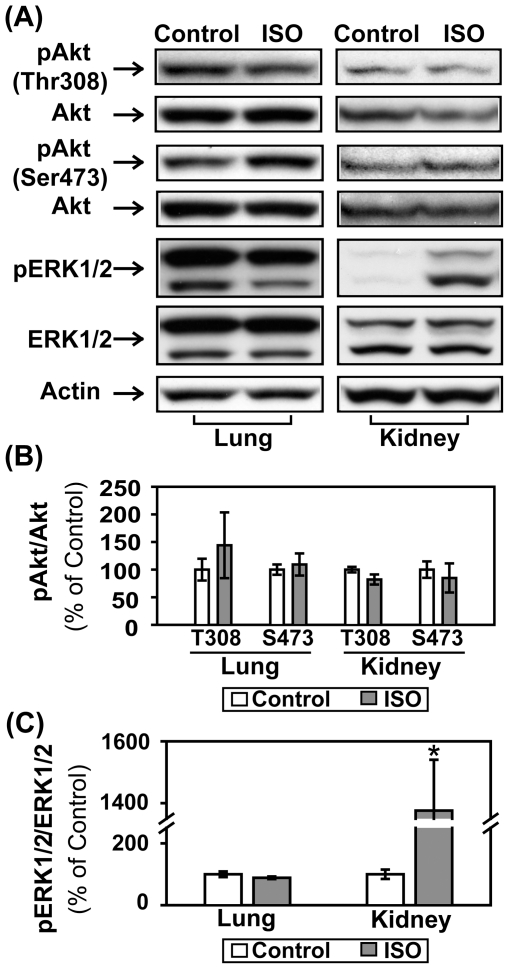
Acute βAR stimulation induced increase in phosphorylation of Akt is cardiac-specific. Adult male C57BL/6 mice were treated with vehicle control (saline) or isoproterenol (ISO, 1.25 mg/kg, i.N) for 30 min. (A) Representative Western blot analyses were performed on lung or kidney tissue lysates with antibodies against phospho-Akt (Thr308), phospho-Akt (Ser473), total Akt, phospho-ERK1/2 (Thr202/Tyr204) and total ERK1/2. (B, C) The bar graphs show the densitometric scanning results from two seperate experiments (n = 6). Data are normalized with individual total protein levels and represent means ± S.E. of percent change in protein phosphorylation relative to that of vehicle control. *, p<0.05 versus vehicle control.

**Figure 5:**
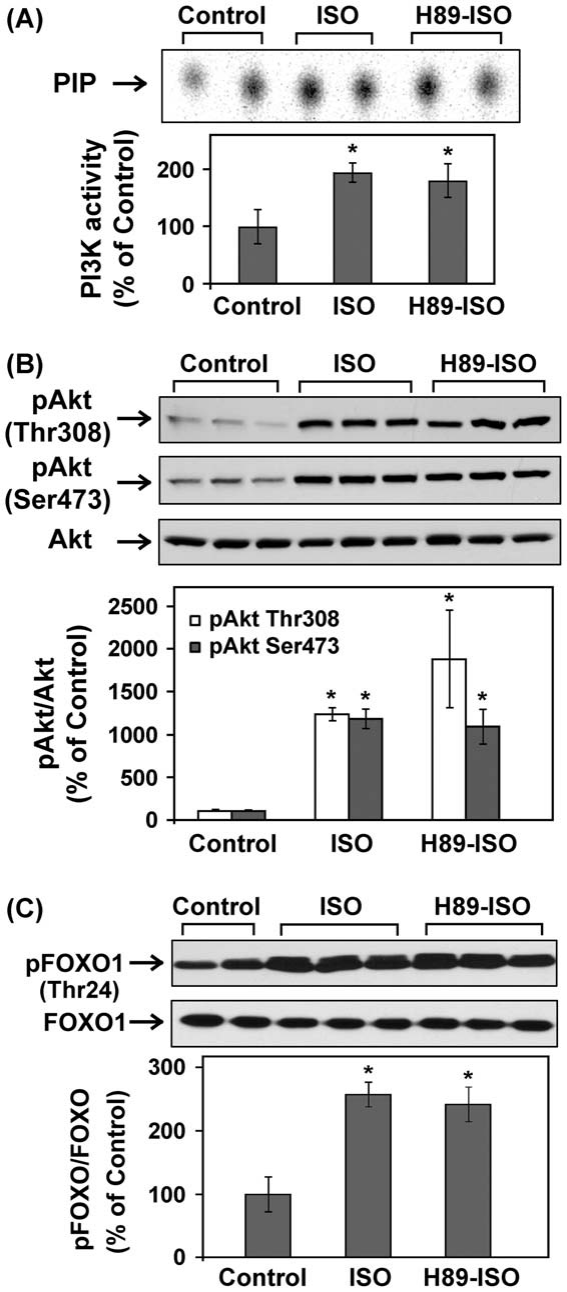
Acute βAR stimulation induced-transactivation of PI3K signaling pathway is PKA-independent. Adult male C57BL/6 mice were pretreated with vehicle (5% DMSO) or H-89 (20 mg/kg, i.p.) for 30 min before treatment with control (saline) or isoproterenol (ISO, 1.25 mg/kg, i.p.) for 30 min. (A) In vitro lipid kinase assay was performed as described. PIP, the phosphorylated end-product. The bar graph shows the densitometric scanning results of the measurement of PI3K activities in the control, ISO-treated and H-89 + ISO-treated mice (n = 4). (B, C) Representative Western blot analyses were performed on LV tissue lysates with antibodies against phospho-Akt (Thr308), phospho-Akt (Ser473), phospho-FOXO1 (Thr24), total Akt and total FOXO. The bar graphs show the densitometric scanning results. Data are normalized with individual total protein levels and represent means ± S.E. of percent change in protein phosphorylation relative to that of the control. *, p<0.05 versus the control.

C57BL/6 mice were treated with saline or ISO for 30 min. Left ventricular tissue lysates were subject to PI3K assay and Western blotting. Acute ISO treatment increased phosphotyrosine-associated PI3K activity, Akt phosphorylation (Thr308 and Ser473), and ERK1/2 phosphorylation in adult mice (Fig. 1A,B,C). Pretreatment with LY294002, a potent PI3K inhibitor, abolished βAR stimulation-induced activation of PI3K and Akt, confirming the direct involvement of PI3K (Fig. 1D,E). In a subset of experiments, mice were treated with ISO for various time periods shorter (15 min) or longer than 30 min (1, 2, 4, or 6 hr). At all time points, ISO treatment significantly increased Akt phosphorylation (Fig. 2). The biggest increase, however, was found within the first hour following ISO treatment and tapered off with time. The 30-mim ISO treatment time point was then used for the experiments described below to examine the acute effect of βAR stimulation. We next further established the role of βAR in the transactivation of PI3K/Akt. The effects of ISO on PI3K/Akt were completely abrogated when the same treatment was performed on age- and gender-matched β_1_/β_2_ DKO mice (Fig. 3A/B). Acute βAR stimulation induced increase in ERK1/2 phosphorylation was also abrogated in β_1_/β_2_ DKO mice (Fig. 3C). To determine if the crosstalk is cardiac-specific, Western blotting was also performed using lung, kidney and liver tissue lysates from control or ISO-treated mice. Acute βAR stimulation did not increase Akt phosphorylation in the lung, kidney (Fig. 4A,B) or liver (data not shown). Interestingly, acute βAR stimulation significantly increased the phosphorylation of ERK1/2 in the kidney but not in the lung (Fig. 4A,C). It is well established that βAR stimulation can induce cellular effects via the activation of G proteins/cAMP/PKA signaling pathway. It is not known, however, if the cAMP/PKA cascade is required for the crosstalk between acute βAR stimulation and PI3K in vivo. Our previous study based on in vitro model suggests that cAMP/PKA pathway may not be needed for such crosstalk [7]. To investigate this, mice were pretreated with H-89, a PKA inhibitor, for 30 min before injection with ISO for 30 min. As expected, ISO treatment significantly increased cardiac PI3K activity and phosphorylation of Akt and FOXO1(Thr24) which were not affected by pretreatment with H-89 (Fig. 5).This suggests that the cAMP/PKA signaling pathway is not directly involved with βAR stimulation-mediated activation of PI3K signaling pathway. Taken together, these data provide solid evidence for a crosstalk between βAR and PI3K signaling pathway in adult mouse heart.

### Acute βAR stimulation- induced increase in phosphorylation of Akt and ERK1/2 is β_2_AR-specific

**Figure 6:**
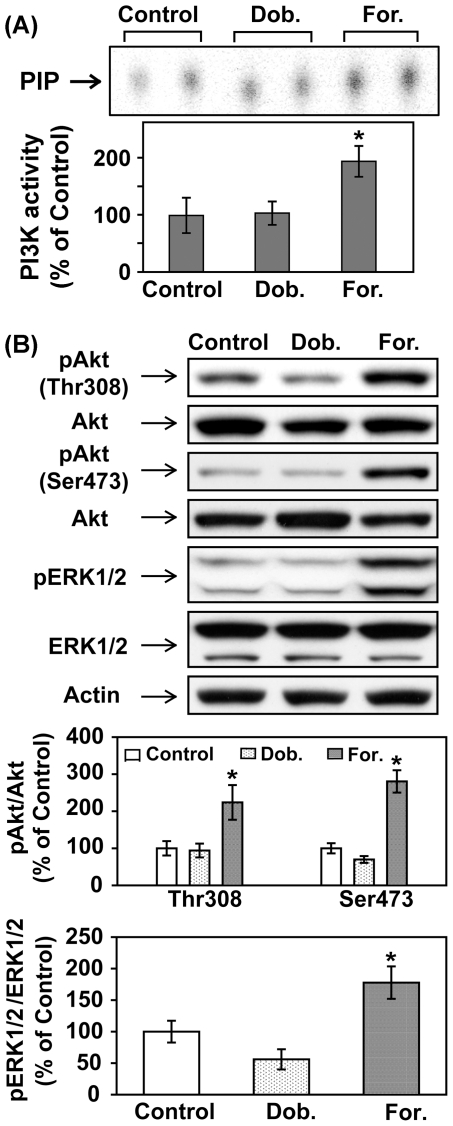
Acute βAR stimulation induced increase in phosphorylation of Akt and ERK1/2 is β2AR-specific. C57BL/6 mice were treated with vehicle control, dobutamine (Dob., 1.7 mg/kg, i.p.), or formoterol (For., 2.1 mg/kg, i.p.), for 30 min. (A) In vitro lipid kinase assay was performed as described except with higher amount of LV lysates (1 mg). PIP, the phosphorylated end-product. The bar graph shows the densitometric scanning results of the measurement of PI3K activities in the control, Dob.-treated and For.-treated mice (n = 4). (B) Representative Western blot analyses were performed on left ventricular lysates with antibodies against phospho-Akt (Thr308), phospho-Akt (Ser473), total Akt, phospho-ERK1/2 (Thr202/Tyr204) and total ERK1/2. The bar graphs show the densitometric scanning results from two seperate experiments (n = 5). Data are normalized with individual total protein levels and represent means ± S.E. of percent change in protein phosphorylation relative to that of vehicle control. *, p<0.05 versus vehicle control.

**Figure 7:**
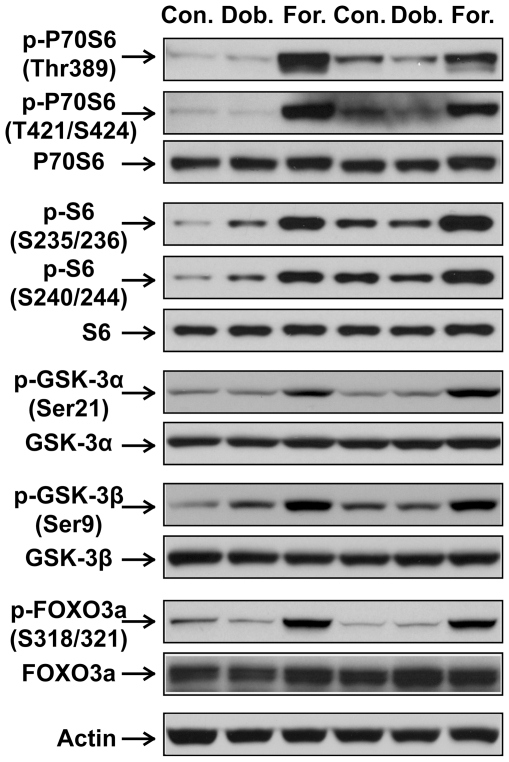
Acute β2AR stimulation induced increases in phosphorylation of P70S6, S6, GSK3α, GSK3β and FOXO3a. C57BL/6 mice were treated with vehicle control, dobutamine (Dob., 1.7 mg/kg, i.p.), or formoterol (For., 2.1 mg/kg, i.p.), for 30 min. Shown are representative Western blots performed on left ventricular lysates with antibodies against phospho-P70S6K (Thr389), phospho-P70S6K (Thr421/Ser424), phospho-S6 (Ser235/236), phospho-S6 (Ser240/244), phospho-GSK-3α (Ser21), phospho-GSK-3β (Ser9), phospho-FOXO3a (Ser318/321). Blots of individual total protein and activ were also included.

As mentioned earlier, both β_1_AR and β_2_AR are expressed in the heart. To assess which subtype of βAR is responsible for ISO-induced increase in phosphorylation of Akt and ERK1/2, mice were treated with saline, dobutamine (a predominantly β_1_AR agonist) or formoterol (a β_2_AR-selective agonist) for 30 min. Acute β_2_AR stimulation with formoterol induced significant increases in PI3K activity (Fig. 6A) and phosphorylation of both Akt (Thr308, Ser473) and ERK1/2 (Fig. 6). In contrast, acute β_1_AR stimulation with dobutamine had no effect (Fig. 6B). In a subset of experiments, we further examined changes in phosphorylation in other downstream signaling factors. Consistent with results shown in Fig. 6, acute treatment with formoterol, but not dobutamine, increased phosphorylation of P70S6 (Thr389 and T421/S424), S6 (S235/236 and S240/244), GSK3α (Ser21), GSK3β (Ser9) and FOXO3a (S318/321) (Fig. 7). These results suggest that the effects of acute βAR stimulation on PI3K signaling pathway and ERK1/2 in adult mouse heart are β_2_AR-specific.

### Acute βAR stimulation increases phosphorylation of other signaling factors downstream of PI3K

**Figure 8:**
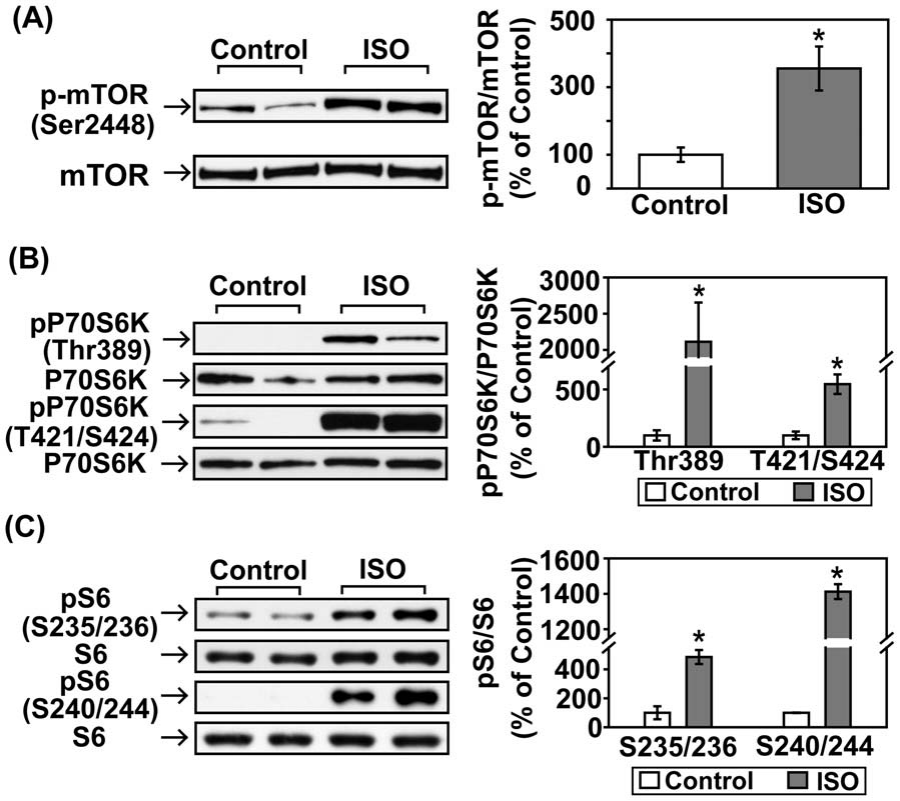
Acute βAR stimulation in vivo induces increases in the phophorylation of mTOR, P70S6K and S6. C57BL/6 mice were treated with vehicle control or isoproterenol (ISO, 1.25 mg/kg, i.p.) for 30 min. Representative Western blot analyses were performed on left ventricular lysates with antibodies against (A) phospho-mTOR (Ser2448) and total mTOR; (B) phospho-P70S6K (Thr389, Thr421/Ser424) and total P70S6K; (C) phospho-S6 (Ser235/236, Ser240/244) and total S6. The bar graphs show the densitometric scanning results from two seperate experiments (n = 6). Data are normalized with individual total protein levels. *, p<0.05 versus vehicle control.

We next examined other signaling molecules downstream of PI3K following the same ISO treatment. The mammalian target of rapamycin (mTOR) is an important downstream effector of PI3K/Akt pathway [29]. Following acute ISO treatment, phosphorylation of mTOR (Ser2448) was markedly increased (Fig. 8A). Phosphorylation of P70S6K (ribosomal protein S6 kinase 1), immediately downstream of mTOR [30], was significantly increased at both the Thr389 and Thr421/Ser424 sites (Fig. 8B). Furthermore, phosphorylation of ribosomal protein S6 (Ser235/236, S240/244), the effector of p70S6K, was also significantly increased after acute βAR stimulation in mouse heart (Fig. 8C).

**Figure 9:**
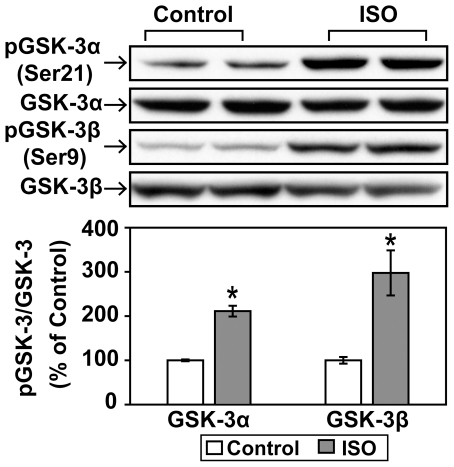
Acute βAR stimulation in vivo induces increases in the phophorylation of GSK-3α and GSK-3β. C57BL/6 mice were treated with vehicle control or isoproterenol (ISO, 1.25 mg/kg, i.p.) for 30 min. Representative Western blot analyses were performed on left ventricular lysates with antibodies against phospho-GSK-3α (Ser21), total GSK-3α, phospho-GSK-3β (Ser9), and total GSK-3β. The bar graphs show the densitometric scanning results from two seperate experiments (n = 6). Data are normalized with individual total protein levels. *, p<0.05 versus vehicle control.

Glycogen synthase kinase-3 (GSK-3) is another critical downstream element of the PI3K/Akt signaling pathway. These kinases are key signaling mediators in myocardial hypertrophy and progressive heart failure [31]. As shown in Fig. 9, acute βAR stimulation induced a significant increase in the phosphorylation of both GSK-3α (Ser21) and GSK-3β (Ser9).

**Figure 10:**
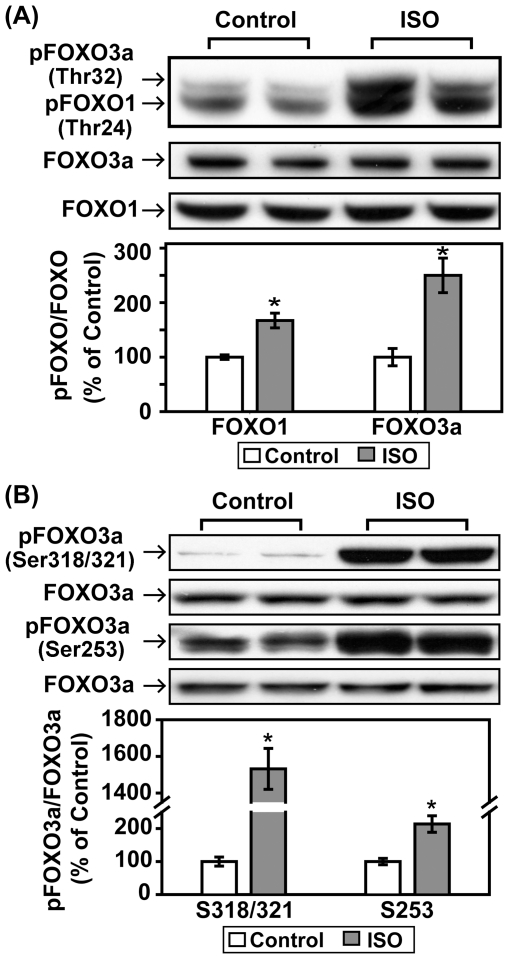
Acute βAR stimulation *in vivo* induces an increase in the phophorylation of FOXO1 and FOXO3a. C57BL/6 mice were treated with vehicle control or isoproterenol (ISO, 1.25 mg/kg, i.p.) for 30 min. Representative Western blot analyses were performed on left ventricular lysates with antibodies against phospho-FOXO1(Thr24)/FOXO3a(Thr32), phospho-FOXO3a (Ser318/321, Ser253), total FOXO1 and total FOXO3a. The bar graphs show the densitometric scanning results from two seperate experiments (n = 6). Data are normalized with individual total protein levels. *, p<0.05 versus vehicle control.

The forkhead box family of transcription factors (FOXOs) is implicated in the regulation of a variety of cellular processes, including the cell cycle, apoptosis, DNA repair, stress resistance, and metabolism. FOXOs proteins are negatively regulated by the PI3K/Akt signaling pathway [32]. We have demonstrated, for the first time, that acute βAR stimulation induced significant increases in the phosphorylation of both FOXO1 (Thr24) and FOXO3a (Thr32, Ser318/321, Ser253) *in vivo* (Fig. 10). Taken together, these data indicate that acute βAR stimulation-induced signaling cascade involves multiple factors downstream of Akt, including mTOR/P70S6K/S6, GSK-3α/β and FOXO1/3a.

### Acute βAR stimulation reduces the mRNA levels of atrgin-1 and MuRF1

**Figure 11:**
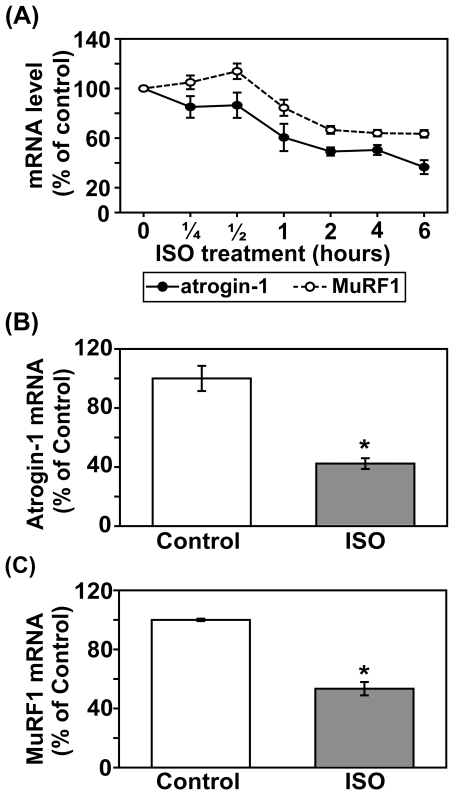
Acute βAR stimulation time-dependently decreases the levels of atrogin-1 and MuRF1 mRNA in mouse heart. Adult male C57BL/6 mice were treated with vehicle control or isoproterenol (ISO, 1.25 mg/kg, I.p.) for up to six hours (A). In a separate experiment, mice were treated with vehicle control or ISO for four hours. The mRNA levels of atrogin-1 (B, n = 3) and MuRF1 (C, n = 3) were determined by real time quantitative PCR using specific TaqMan probes and normalized to GAPDH. The plot lines or bar graphs represent the means ± S.E of percent change in mRNA level relative to that of vehicle control. *, p<0.05 versus vehicle control.

It has been shown that atrogin-1 and MuRF1 are associated with hypertrophy and atrophy processes in skeletal muscle through the PI3K/Akt signaling pathway [33], [34]. To investigate whether cardiac atrogin-1 and MuRF1 are affected by acute βAR stimulation, we examined their mRNA levels by real-time quantitative PCR. As shown in Fig. 11A, βAR stimulation induced a time-dependent reduction of both atrogin-1 and MuRF1 mRNA levels. The trend in reduction in the mRNA levels can be seen as early as one hour following ISO treatment. To confirm these results, mice were treated with saline or ISO for 4 hours. A similar reduction (50%∼60%) in the mRNA levels of both atrogin-1 and MuRF1 was seen in the ISO-treated mice (Fig. 11B/C). This is the first demonstration in mouse heart that βAR stimulation reduces the mRNA levels of atrogin-1 and MuRF1.

## Discussion

In the present study, we set out to elucidate the early signaling events that mediate the effect of acute βAR stimulation in mouse heart. Our results provide convincing *in vivo* evidence for the crosstalk between βAR and PI3K signaling pathway. We demonstrate that the βAR transactivation of PI3K/Akt pathway is β_2_AR-specific and confines to the heart. Many known signaling factors downstream of PI3K, including the mTOR/P70S6K/S6 axis, GSK-3α/β, and FOXO1/FOXO3a, were shown to be involved. Although these cascades have been well investigated, the link between βAR and these signaling pathways has not been systematically characterized *in vivo*, especially in the cardiac context. More important, we demonstrate, for the first time, that atrogin-1 and MuRF1 are affected by acute βAR stimulation in mouse heart.

The levels and activity of the cardiac PI3K signaling pathway are tightly controlled during development. The highest activity is found in late gestation and early postnatal life and then decreases dramatically in older animals [18]. We have *in vitro* and *in vivo* evidence showing that βAR stimulation induces activation of cardiac PI3Kα but not the other isoforms of PI3K [7]. However, the responsible signaling mediators downstream of PI3K after acute βAR stimulation have not been well established. The results of the current study are the first systematical identification of multiple important signaling factors for such βAR-PI3K crosstalk.

Akt is a serine/threonine protein kinase downstream of PI3K. Full activation of Akt requires phosphorylation at both Thr308 [by 3–phosphoinositide–dependent protein kinase 1 (PDPK1)] and Ser473 (by a putative PDPK2) [35], [36]. In mouse heart, acute βAR stimulation increases the phosphorylation of Akt at both the Thr308 and Ser473 sites (Fig. 1B). ERK1/2 is another important signaling effector involved in development of βAR-mediated cardiac hypertrophy [37], [38]. Following acute βAR stimulation, ERK1/2 is also activated (Fig. 1C). It is likely, that the signaling cascades responsible for βAR-induced cardiac hypertrophy are not confined to PI3K and ERK1/2. Moreover, it is possible that there are different mechanisms underlying induction of hypertrophy, depending on the pathological stimuli [39].

β_1_/β_2_ DKO mice are viable, fertile, normal in size and do not display any gross physical or behavioral abnormalities [40]. It has been shown that the impact of β_1_/β_2_AR loss on basal physiological functions such as heart rate, blood pressure, or metabolic rate is remarkably modest. Hence, β_1_/β_2_ DKO mice represent a useful model for the study of βAR-modulated cardiac functions *in vivo* [40]. We showed that transactivation of PI3K and the subsequent activation of Akt and ERK1/2 following acute βAR stimulation were completely abrogated in β_1_/β_2_ DKO mice, providing further evidence for the cardiac βAR-PI3K crosstalk *in vivo*.

There is increasing evidence to support a differential role of β_1_AR and β_2_AR in terms of regulation of cardiac function and alterations in cardiac structure [6], [26], [41], [42], [43]. For example, β_1_AR has been shown to mediate pro-apoptotic responses while β_2_AR mediates anti-apoptotic responses in cardiomyocytes [26], [43]. β_2_AR signaling has a lower overall effect on contractility than β_1_AR signaling does [44]. Coupled to Gα_i_, β_2_AR signaling has been demonstrated to induce the recruitment and activation of other signaling molecules, including PI3K/Akt and ERK [5], [22], [44], [45]. We have previously shown βAR stimulation-induced activation of PI3K signaling pathway is β_2_AR-specific in H9c2 cardiomyoblasts, a β_2_AR predominant cell line [7]. In comparison, β_1_AR comprises approximately 70–80% of the total βAR population in healthy heart [42]. Despite these differences of βAR subtype abundance in H9c2 cells and in mouse heart, acute βAR stimulation-induced activation of PI3K signaling pathway appears to be β_2_AR-specific. It is unknown, however, whether or not chronic βAR stimulation-induced cardiac hypertrophy is solely mediated by β_2_AR. It has been reported in cultured rat neonatal cardiomyocytes that βAR-induced hypertrophy is mediated primarily by the β_1_AR subtype [46]. This discrepancy may reflect the fundamental differences between *in vitro* and *in vivo* systems. The *in vivo* data shown here are novel and add new insights to our understanding regarding the characteristics among βAR subtypes.

This crosstalk may be tissue-specific since the same βAR stimulation did not activate Akt in the lungs, kidneys (Fig. 4) or liver. Acute βAR stimulation, however, did activate ERK1/2 in the kidneys (but not in the lungs). These observations are interesting and warrant further future investigation.

Activation of Akt and mTOR plays a crucial part in the regulation of cell growth and proliferation by monitoring nutrient availability, cellular energy levels, oxygen levels and mitogenic signals [29], [47], [48]. One of the most extensively characterized downstream targets of mTOR is P70S6K, which is pivotal to the regulation of protein synthesis, via the phosphorylation and activation of its major effector, ribosomal protein S6 [29], [30]. In the present study, we demonstrated that acute βAR stimulation in adult mouse heart increased the phosphorylation of the whole mTOR/P70S6K/S6 signaling axis at multiple sites. Considering the important roles of growth promotion by the mTOR/P70S6K/S6 signaling axis, it is plausible to reason that chronic βAR activation may lead to cardiac hypertrophy via this signaling pathway.

Akt-mediated phosphorylation of GSK-3 is inhibitory [49]. It is believed that GSK-3 inhibition induced-cardiac hypertrophy is independent of the mTOR pathway [31], [50]. It has been shown that GSK-3β inactivation occurs in response to βAR agonists [20]. More recent studies also indicate that expression of inactivation-resistant GSK-3α/β isoforms avert the pathological hypertrophy caused by chronic ISO stress [51]. In isolated C57BL/6 mice hearts, acute ISO exposure resulted in significant GSK-3β, but not GSK-3α, phosphorylation. In most previous studies, particular attention has focused on GSK-3β because it is regarded as the predominant isoform, but increasingly GSK-3α has been considered to play equally important roles. For example, it has been shown that up-regulation of GSK-3α inhibited cardiac growth and pressure overload-induced cardiac hypertrophy which is mediated through inhibition of ERK [52]. Our data provide strong evidence that acute βAR stimulation induces phosphorylation (inhibition) of both GSK-3α and GSK-3β *in vivo*.

Phosphorylation of FOXOs by Akt is inhibitory and leads to nuclear exclusion and the inhibition of the forkhead transcriptional program [53]. Three members of the FOXOs, FOXO1, FOXO3a, and FOXO4, have been implicated in regulating diverse cellular functions including differentiation, metabolism, proliferation, and survival [32], [54]. In cardiomyocytes, it has been established that FOXO1 and FOXO3a regulates apoptotic responses [55] and cell size [56], respectively. Recently, FOXOs have also been shown to promote atrophy in skeletal muscle [34], [57]. It is, however, not known if the regulation and function of FOXOs in the heart are under the control of βAR. Our study provides evidence, for the first time, that acute βAR stimulation results in significant inhibition of both FOXO1 and FOXO3a in mouse heart.

It has been extensively investigated that atrogin-1 and MuRF1 are common mediators in several models of skeletal muscle hypertrophy and atrophy. The levels of atrogin-1 and MuRF1 are regulated by the growth factor/Akt signaling axis through direct transcriptional regulation by FOXOs [33], [34], [57]. β_2_AR–agonists has also been shown to increase skeletal muscle strength in normal volunteers [58] and in a small number of patients [59]. In a cardiac context, atrogin-1 and MuRF1 have been demonstrated to inhibit cardiac hypertrophy [56], [60], [61]. It was also shown that atrogin-1 and MuRF1 regulate cardiac myosin-binding protein C which is related to familial hypertrophic cardiomyopathy [28]. Moreover, recent clinical and experimental studies have suggested that βAR activation may prevent LV atrophy during LV unloading [11], [12], with the mechanism unknown. However, it has not been reported whether or not atrogin-1 and MuRF1 are involved in the βAR stimulation-induced cardiac hypertrophy and/or the anti-atrophic effect. Our finding is the first to demonstrate the link from βAR to atrogin-1 and MuRF1 in the heart, presumably via PI3K/Akt/FOXOs. Further study of this crosstalk should yield useful insights that may have important clinical implications.

**Figure 12:**
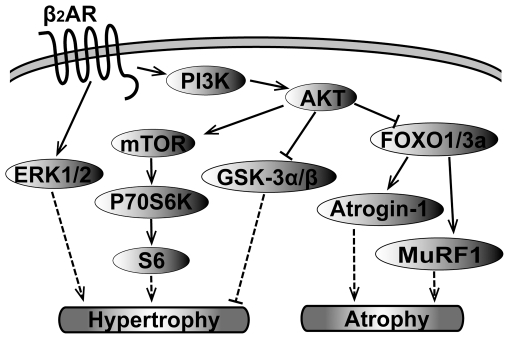
A hypothetical signaling cascades for the crosstalk between β2AR and PI3K/Akt pathway *in vivo*. Activation of ERK1/2-related signaling and the mTOR/P70S6K/S6 axis favors the progression of cardiac hypertrophy. Akt-induced phosphyrylation of GSK-3α/β inhibits its activity, thereby nullifying its anti-hypertrophic effect. Akt-induced phosphorylation of FOXOs also inhibits ist activity, negating its effects on activation of atrogin-1 and MuRF1, which results in decreased protein breakdown (atrophy). These multiple signaling scenarios likely contribute to the pathogenesis of cardiac hypertrophy and/or the anti-atrophic effect following sustained βAR stimulation. “→” represents activation, “⊣” represents inhibition. Solid lines represent data supplied by our study, and dotted lines represent hyperthetical schematics.

In summary, our data suggest that crosstalk between βAR and PI3K signaling pathway activates multiple downstream signaling effectors and cascades *in vivo*. Our data support a model depicted in Fig. 12, where acute stimulation of the β_2_AR activates PI3K, which affects the activities of ERK1/2, Akt and its downstream signaling cascades, including mTOR/P70S6K/S6 axis, GSK-3α/β, FOXOs, atrogin-1 and MuRF1. These data provide mechanistic and signaling insights for the pathophysiological consequences induced by chronic βAR stimulation.
